# Distribution Characteristics and Ecological Risk Assessment of Tetracyclines Pollution in the Weihe River, China

**DOI:** 10.3390/ijerph15091803

**Published:** 2018-08-22

**Authors:** Ying Li, Jie Fang, Xiaoyu Yuan, Yangyang Chen, Hongbin Yang, Xiaohua Fei

**Affiliations:** 1 Key Laboratory of Subsurface Hydrology and Ecological Effects in Arid Region, Ministry of Education, Chang’an University, Xi’an 710054, China; 15291192057@163.com (Y.L.); 18792975240@163.com (J.F.); m15129037687@163.com (X.Y.); 18291960210@163.com (Y.C.); 2016229036@chd.edu.cn (H.Y.); 2 Xi’an Center of Geological Survey, China Geological Survey, Xi’an 710054, China

**Keywords:** tetracycline antibiotics, surface water, sediment, Weihe River

## Abstract

To examine the residual and distributions of tetracycline antibiotics in the Weihe River, SPE-UPLC (solid phase extraction-ultra performance liquid chromatography with UV-Vis detection) was employed to analyze the oxytetracycline (OTC), chlortetracycline (CTC), and minocycline (MC) of 41 surface water and 35 sediment samples collected from main streams, tributaries, and main sewage outlets. The results showed that: (1) The order of residual levels of tetracycline antibiotics in water and sediment from high to low was the following: OTC > CTC > MC., considering the water solubilities are 313 mg/L, 630 mg/L, and 50200mg/L and octanol water partition coefficients (K_ow_) are 7.94, 4.16, and 1.12 for OTC, CTC, and MC, respectively. Thus, the distribution of antibiotics was not only related to the basic properties of antibiotics, but also some environmental factors. The concentrations of OTC in water and sediment were in the range of 1.56–87.89 ng/L and 6.13–45.38 ng/g (mean value of 16.13 ng/L and 20.60 ng/g), respectively; while CTC was 1.07–26.78 ng/L and 6.17–32.29 ng/g (mean value of 4.96 ng/L and 14.48 ng/g), respectively; and MC was 0.28–12.35 ng/L and 4.80–29.74 ng/g (mean value of 1.70 ng/L and 12.96 ng/g), respectively. There were maximum concentrations in all sewage outlets. Compared with other areas in China, tetracyclines residual in the Weihe river were at a medium level; (2) in spatial distribution, the levels of tetracyclines in water and sediment from the middle and upper reaches were higher than the lower reaches. Meanwhile, the sewage outfalls and livestock farm waste water discharge appeared to be the main sources of tetracycline antibiotics in the Weihe River; (3) ecological risk assessment revealed that in main streams and tributaries, OTC and CTC may be at a low ecological risk level; while in sewage outfalls, they may represent a medium ecological risk level.

## 1. Introduction

Antibiotics can selectively impair or influence the function of other organisms at low concentrations [1]. Antibiotics are an important pollutant in water [2]. China is the largest producer and user of antibiotics in the world. Based on the survey, the total usage for the 36 chemicals was 92700 tons in 2013, and eventually, 53800 tons of them entered into the environment [3]. Because antibiotics were not completely degraded by organism metabolism, 25–75% in the form of a matrix or its metabolic product were released out of the body to the environment, which posed a serious threat to the ecological environment and human health [4].

Tetracyclines (TCs) are considered as one of the most widely used antibiotics because of their advantages of a broad spectrum, high quality, and low price [5]. In recent years, scholars have paid much attention to the residue of TCs in water and sediment in China. Jiang et al. (2014) studied 16 kinds of antibiotics in water and sediment of Wangyang River, Anhui Province [6]. The results showed that the concentrations of oxytetracycline and chlortetracycline in water were as high as 3.6 × 10^5^ ng/L and 6.9 × 10^4^ ng/L, respectively, and the concentrations of oxytetracycline in sediment were 1.6 × 10^5^ ng/g. Xu et al. (2014) found that the concentrations of oxytetracycline and chlortetracycline in Taihu Lake were 44.2ng/L and 69.7ng/L, respectively [7]. Chen et al. (2015) detected high concentrations of TCS in the South Gulf region, and they found that oxytetracycline may pose a high risk to aquatic organisms [8]. A national reconnaissance of 139 streams in the USA showed that the maximum detected concentration of oxytetracycline is 340 ng/L [9]. A study that monitored the veterinary medicines in water bodies in the UK reported that OTC is present at maximum concentrations of 4490 ng/L in the surface water environment [10]. Therefore, it is of great significance to study the contents, spatial distribution, and ecological risk of TCs in water and sediment in densely populated areas.

The Weihe River, as the largest tributary of the Yellow River, mainly flows through Tianshui, Baoji, Xianyang, Xi’an, and Weinan areas in China. The region is well developed in industry and agriculture. Therefore, the pollution of organochlorine pesticides and heavy metal pollution in the Weihe River has attracted much attention [11,12]. Despite the long-term use of antibiotics in the region, there are few reports on the pollution situation of the soil and water environment. Therefore, the objectives of this study were to: (1) investigate the occurrence of three kinds of antibiotics in water and sediment in the Weihe River; (2) evaluate the spatial distribution and the sources of the antibiotics based on the correlations between environmental variables and antibiotic concentrations; (3) assess the ecologic risk of the antibiotics to the aquatic organisms according to the calculated risk quotients (RQs).

## 2. Materials and Methods

### 2.1. Instruments and Reagents

Oxytetracycline (OTC), chlortetracycline (CTC), and minocycline (MC) were purchased from Sigma Company. The methanol, acetonitrile, and oxalic acid used were High Performance Liquid Chromatography (HPLC) grade. All other reagents used were analytical grade purchased from Tianjin Kemiou Chemical Reagent Co., China.

### 2.2. Study Area and Sample Collection

The Weihe River is a river in west-central China and is the largest tributary of the Yellow River. The source of the River is close to Weiyuan county in Gansu province. The Guanzhong section of the Weihe River flows through cities such as Baoji, Yangling, Xianyang, Xi’an, and Weinan. It finally merges into the Yellow River at Gangkou town, Tongguan county. The length of the river is 818 km and the area drained covers 135,000 km^2^. Because of the intensive human activities in the Guanzhong section, the pollution situation in this area is severely induced by the soil and water loss of farmland, as well as the discharge of industrial wastewater and domestic sewage, which have seriously influenced and restricted the sustainable development of the region.

**Figure 1:**
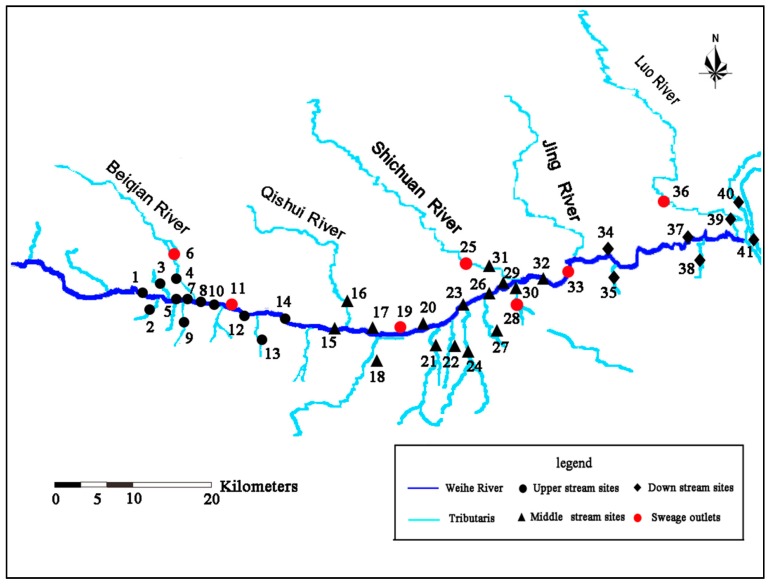
Sampling sites of the Weihe River (water samples and sediment samples for 1–41).

We selected 41 sampling sites along the Weihe River and its tributaries in order to cover the whole aquatic system in this area (Figure 1). The sampling location’s latitudes ranged from 107°2′ to 110°17′ and longitudes ranged from 34°20′ to 34°41′, and detailed information of the sampling sites is presented in Table S1 (Supplementary materials). In this study, 1 L water samples were collected using a cleaned brown glass bottle and about 1 kg composite sediment samples (0–5 cm deep) were collected with a stainless-steel static gravity corer at every sampling site. In total, 41 water samples and 35 sediment samples were collected (some sampling sites cannot obtain sediment samples). All the equipment used for sample collection, transportation, and preparation was cleaned with purified water before every sampling to prevent tetracycline antibiotics contamination.

### 2.3. Sample Extraction and Analysis

The samples extraction and analysis technique followed the reported method of Xu and Wu [13], with some changes. Water samples (100 mL) were passed through the glass fiber membrane (0.45 μm) at a flow rate of 5 mL/min using a vacuum. All water samples were extracted and purified using a solid phase extraction (SPE) system. The Oasis HLB columns were first conditioned with 3 mL of methanol, and then 3 mL of deionized water (pH = 3), followed by 3 mL of methanol aqueous solution. Following extraction, the columns were eluted with 6 mL of methanol. Then, the extracts were concentrated to 1 mL by a rotary evaporator prior to HPLC analysis.

To make the samples more uniform and representative, all the sediment samples were freeze-dried, homogenized, and passed through a 150-μm sieve. EDTA buffer solution (20 mL, 0.1 mol/L) was added [14] to each sample (2 g) and the samples were then extracted by ultrasonic extraction for 10 min. Following this, a 10 mL aliquot of the supernatant was extracted by the same method employed for water samples indicated above.

A Waters ACQUITY Ultra Performance Liquid Chromatography (UPLC) H-Class with an ultraviolet/visible spectrophotometer detector and C18 1.7 mm 2.1 × 100 mm column was used for the quantification of OTC, CTC, and MC in solution. Column temperature was 40 ± 0.1 °C and sample 10 ± 0.1 °C. Injection volumes of 5 μL were used, where the mobile phase contained effluent A (0.01 mol/L oxalic acid): B (acetonitrile): C (methanol) = 70%: 15%: 15%, with a flow rate of 0.1 mL/min. OTC, CTC, and MC were all measured at 265 nm.

### 2.4. Quality Analysis and Quality Control

All analytical operations were conducted under strict quality control guidelines (ISO 5725). Procedural blank and spiked samples consisting of all reagents were run to check for interference and cross-contamination. Briefly, the spiking of the samples started at the beginning of the experiment, arbitrary duplicate water and sediment samples were chosen, one of the relevant water and sediment samples received a certain amount of three standard antibiotics, and other steps of the samples followed the samples analysis process described in 2.3. The limits of detection for three kinds of antibiotics were determined as analyte concentrations in a sample that gives rise to a peak with a signal-to-noise ratio (S/N) of 3. The method detection limits (MDLs) for three kinds of antibiotics in water were in the range of 0.12–0.27 ng/L. The spiked recoveries in water ranged from 79% to 113%. The MDLs for three kinds of antibiotics in sediment were in the range of 0.45–0.79 ng/g (dw). The spiked recoveries in sediment ranged from 62% to 92%. The relative standard deviation was 15–22% for water samples and 20–27.5% for sediments.

### 2.5. Risk Assessment Method

According to the European technical guidance document (TGD) on risks assessment [15], which can propose the ecological safety threshold of relevant pollutants and is also an important basis for the ecological risk assessment and management of these chemicals, the potential ecological risks of the detected antibiotics were assessed using the risk quotients (RQs) approach [16] and the RQS value was obtained by Equation (1).

(1) $$\;\mathrm{RQS}\;=\;\frac{\mathrm{MEC}}{\mathrm{PNEC}}\;$$

where MEC is the measured environmental concentration, and PNEC is the predicted non-effect concentration for each contaminant. PNEC is the quotient of the lowest no-observed-effect concentration (NOEC) for the most sensitive species with an appropriate assessment factor (AF). Given the insufficient NOEC data for most compounds, PNEC was extrapolated from the acute toxicity data or chronic toxicity data of compounds through the division of an appropriate AF. The AFs of 100 and 1000 for chronic and acute toxicity were used [17] in the following equation:

(2) $$\;\mathrm{PNEC}\;={\mathrm{EC}}_{50}({\mathrm{LC}}_{50})/1000\;$$

Or,

(3)  PNEC =chv/100 

where EC_50_(LC_50_) and chv represent acute toxicity and chronic toxicity, respectively.

The RQ values for the target antibiotics calculated from PNEC were determined by the relevant most sensitive species [18]. To better distinguish the ecological risk levels, the RQ values were classified into three risk levels (0.01–0.1: low risk level; 0.1–1: medium risk level; >1: high risk level) [16].

## 3. Results and Discussion

### 3.1. Pollution Characteristics of TCs in the Weihe River

#### 3.1.1. Pollution Characteristics of TCs in Water

The detailed concentrations of three antibiotics in water are listed in Table S3. The detectable rates of three kinds of tetracycline (OTC, CTC, and MC) in 41 water samples were 100%, indicating that this type of antibiotic is widely distributed in water of the Weihe River. As shown in Table 1, the detectable concentration of OTC in water was high, being within 1.56–87.89 ng/L, and the mean concentration was 16.13 ng/L. The highest concentration was observed at Dazhang Temple outlet (S11), which was located in the hoggery region in Yangping, Baoji. OTC was used as a veterinary drug and feed additive for cultivation. Due to the low absorbency of antibiotics in animals, considerable levels of OTC were likely excreted into the sewage stream, together with excrement and urine [4]. Compared with other waters in China, the OTC concentration was far lower than that in the Wangyang River [6], Huangpu River [19], Xiang River [20], and Taihu Lake [7]; higher than that in the Jiulongjiang Estuary [21], Boyang Lake [14], Bohai Gulf [22], and Changjiang Estuary [23]; and equal to the residual level observed in the intertidal zone of the Yellow River Delta [24]. The concentration range of CTC and MC was 1.07–26.78 ng/L and 0.28–12.35 ng/L, and the mean concentration was 4.96ng/L and 1.70 ng/L, respectively. The highest concentration point was near XianYang Iron Bridge (S25), which was located in the XianYang urban area and receives domestic sewage of citizens. This is consistent with the conclusion that high-concentration antibiotics for human use can usually be detected in domestic sewage as CTC and MC are used more for medication [25]. Thus, densely inhabited regions tend to be severely afflicted regions of antibiotics for human use. Compared with other waters, the residual concentration of CTC was lower than that in Taihu Lake [7] and Wangyang River [6], and higher than that in Boyang Lake [14], Changjiang Estuary [23], Huangpu River [19], and Xiang River [20]. Generally speaking, the residual of TCs in water of the Weihe River was at a medium level (Table 2). Among the three tetracyclines, the residual level of OTC was far higher than that of the other two, which indicated that OTC served as the main tetracyclines pollutants in water of the Weihe River, considering that the higher OTC concentration came from the mixture of sewage outlets of human domestic discharge and livestock farms (Dazhang Temple outlet (S11), Xingping Wastewater Treatment Plant (S19) and the outlets of Xianyang Iron Bridge (S25)), while only human domestic sewage discharge (S6, S28, S33, and S36) did not generate the obvious high increase of OTC concentration, so we speculated that the OTC source was mainly livestock farm discharge.

#### 3.1.2. Pollution Characteristics of TCs in Sediment

The detailed concentrations of three antibiotics in sediment are listed in Table S3. The detectable rates of three tetracyclines (OTC, CTC, and MC) in sediment were 100%. As shown in Table 1, the concentration range was 6.13–45.38 ng/g, 6.17–32.29 ng/g, and 4.80–29.74 ng/g, and the mean concentration was 20.60 ng/g, 14.48 ng/g, and 12.96 ng/g, respectively. The highest concentration point occurred at Dazhang Temple outlet (S11), near the outlet of Baoji sewage treatment plant (S6) and outlet of Xingping sewage treatment plant (S19), which indicated that the residual level of TCs in sediment was largely influenced by TCs concentration in sewage at outlets. Compared with the TCs content in the sediment of other regions, the OTC level in sediment from the Weihe River was lower than that in Bohai Gulf [22], Wangyang River [6], and Tai Lake [7], and higher than that in Changjiang Estuary [23], Huangpu River [11], and Yellow River [26]. CTC content was lower than that in Tai Lake [7] and Wangyang River [6], and higher than Changjiang Estuary [23], Huangpu River [19], Hai River, and Liao River [26]. Thus, the general concentration of TCs in sediment from the Weihe River was at a medium level (Table 2). The order of the concentration in sediment was OTC> CTC> MC. This may be because of the strong adsorptivity of OTC in particulate matter and sediment. In addition, Liu et al. (2012) found that the degradation rate of CTC in the same type of soil is significantly that of OTC [27], which may be another reason for the relatively higher residual level of OTC in sediment.

### 3.2. Spatial Distribution Characteristics and Source Analysis of TCs in the Weihe River

#### 3.2.1. Spatial Distribution Characteristics of TCs in Water

**Figure 2:**
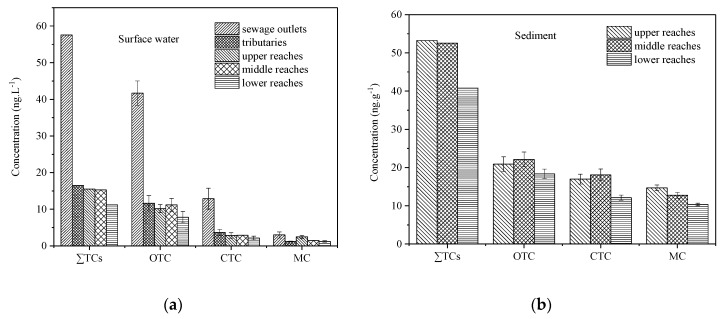
Spatial distribution of TCs in (**a**) surface water and (**b**) sediment from the Weihe River.

Figure 2a shows the distribution of three tetracyclines in main streams, tributaries, and sewage outlets of the Weihe River and their spatial variation trend. It was observed that the concentration of three tetracyclines in water was the same in the upper reaches, middle reaches, and lower reaches of main streams of the Weihe River or even tributaries, while the concentration at outlets was about three times higher than the average of main streams, suggesting that the sewage outlets are an important source for pollution of tetracyclines. This was consistent with the research result of Wen et al. for Daliao River [28]. In terms of river scale, the total concentration of tetracyclines (∑TCs = OTC + CTC + MC) gradually lowered from the upper reaches to the lower reaches. The concentration distribution of OTC and CTC was middle reaches > upper reaches > lower reaches, which may be because the middle reaches region of the Weihe River was located in the periphery of Xi’an urban area and the population was dense there, while the dense population unavoidably increased the use of antibiotics. Meanwhile, the developed open aquaculture increased the consumption of antibiotic OTC, leading to the accumulation of antibiotics that drained into the environment. The concentration distribution of MC was upper reaches > middle reaches > lower reaches, which may be because the particulate matter in lower reaches sediment became smaller, water velocity slowed down, and the adsorption of deposition for minocycline was enhanced. In addition, those three antibiotics concentrations at the upper or middle reaches were higher than the lower reaches, largely due to the fact that two large-scale tributaries, the Jing River and the Luo River, with remittance in the lower reaches area (Figure 1), displayed an obvious dilution effect for the contaminate. Of course, contrary to the upper or middle reaches area, the small population in the lower reaches can also obviously decrease the emitted antibiotics on the other hand.

#### 3.2.2. Spatial Distribution Characteristics of TCs in Sediment

As shown in Figure 2b, the sum of three tetracyclines (OTC, CTC, and MC) in sediment from the upper reaches to lower reaches takes on the trend of a gradual decrease. This may be related to the development degree of agriculture, aquaculture, and concentration of the population along the Weihe River. The concentration distribution of OTC and CTC was middle reaches > upper reaches > lower reaches, and for MC, it was upper reaches > middle reaches > lower reaches. For the three tetracyclines, the concentration distribution in the middle reaches and upper reaches was higher than that in the lower reaches. As well as the properties of antibiotics, the residual of antibiotics in sediment was also influenced by environmental factors such as the ionic strength, temperature, TOC, and particle properties etc. [29]. For example, the concentrations of three antibiotics at S26 sampling sites were higher than those of S23. They are geographically close, while the concentrations were apparently discrepant, maybe because the particle size at S26 was smaller than S23, with the former fine sand content being 43.5% and the latter being 32.6%, so more fine sand content was more favorable for antibiotics adsorbed onto it. Furthermore, the middle reaches and upper reaches developed quickly and had a dense concentration of population, leading to a large consumption of antibiotics. Meanwhile, it likely that the large adsorptive capacity in middle reaches and upper reaches sediment and combined pollution of antibiotics and other pollutants in sediment resulted in a change of enzyme activity, thereby influencing the survival and development of microbes, which slowed down the degradation of antibiotics [30]. However, other combined pollutants in lower reaches suppressed the adsorption of antibiotics in sediment [31]. As a whole, the three tetracyclines showed similar distributional characteristics in sediment and water, except for MC. The differences yielded the properties divergence of MC with two other antibiotics, as well as the usage habit of the pharmaceuticals.

#### 3.2.3. Source Analysis

**Figure 3:**
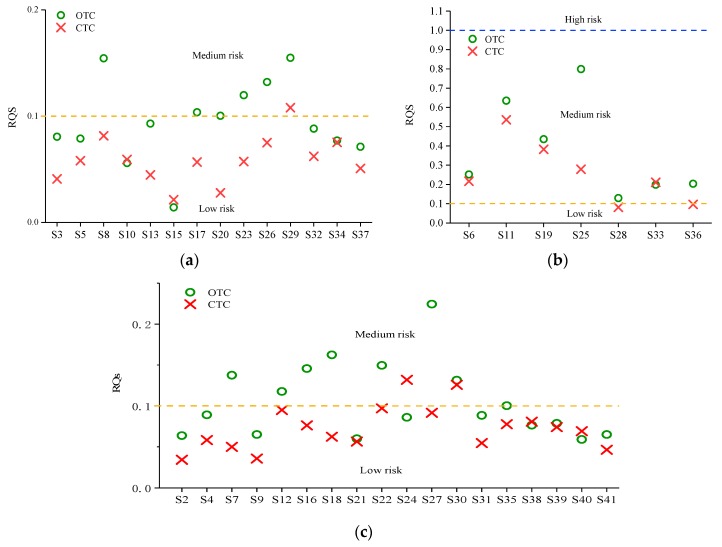
RQs values of OTC and CTC in the (**a**) main streams, (**b**) sewage outlets, and (**c**) tributaries.

The tributaries and sewage outlets are both sources of replenishment of water for main streams of the Weihe River and input sources of pollutants. As shown in Figure 3, the concentration residual of three tetracyclines (OTC, CTC, and MC) in tributaries was the same as that in main streams, while that at sewage outlets was far higher than that in main streams. Thus, in two input sources, the afflux of tributaries would not influence the residual level of tetracyclines in the Weihe River, while the high concentration TCs at sewage outlets would increase the residual in the Weihe River. Thus, it was conjectured that the sewage outlets may be an important input source of TCs in the Weihe River, which was similar to the research result of Chen [19] and Qin [28]. Meanwhile, it was observed from Table 1 that the OTC concentration in water was about 4.5 times the sum of CTC and MC concentration and was far higher than the residual of CTC and MC, which may be because OTCs were widely applied to medical treatment and used as an additive to promote animal growth, while the other two were more frequently used as therapeutic drugs with a consumption far lower than that of OTC. In addition, the typical higher concentrations of the three antibiotics were 69.86 ng/L for OTC, 26.78 ng/L for CTC, and 12.35 ng/L for MC (Table S3), exhibited at Dazhang Temple outlet (S11), which is the sewage outlet of a pig farm. Thus, the waste water from the livestock and poultry industry may also be an important source of TCs in water. The concentration difference among the three tetracyclines in sediment was smaller than that in water, which may be related to the degradation of antibiotics in sediment. Generally speaking, it can be assessed that the TCs in the Weihe River were mainly from waste water effluent from sewage outlets and surrounding livestock and poultry farms.

### 3.3. Ecological Risks Assessment of TCs in the Weihe River

The toxicity data of antibiotics for the most sensitive species is presented in Table 3, and the RQ values of TCs in main streams, tributaries, and sewage outlets calculated using the risk quotient method (no analysis on MCs due to a lack of toxicity data) is showed in Figure 3. It shows that all the samples of OTC and CTC can pose a medium or low risk to the relevant sensitive species (*Pseudokirchneriella subcapitata* and *M. aeruginosa*), while no high risk. In main streams, 37.5% of water samples of OTC posed a medium risk, while the rest posed a low risk. Only 6.25% of CTC in water samples caused a medium risk, and the rest were low risk. In tributaries, 38.8% and 22.2% of samples for OTC and CTC may create a medium risk level, whilst others may cause a low risk. In sewage outlets, all the samples of OTC, while 85.7% of CTC, may lead to a medium risk level. 

The evaluation result showed that most of the samples of OTC and CTC may cause low risk in main streams and tributaries, and hence possessed a tiny influence on the ecological environment. At sewage outlets, most of the sample points for OTC and CTC may cause a medium risk, indicating that the ecological risk of TCs at sewage outlets was relatively high. The residual antibiotics in water may have a certain acute or chronic toxic effect on the aquatic lives, and the long-term residual of antibiotics may also stimulate the pathogenic bacteria to produce drug resistance, which is destined to influence the original stable ecosystem [32]. Harrison et al. [33] also reported that the large usage of antibiotics in the breeding industry may cause resistant genes in livestock, poultry, and fish, and even cause harm to human health by the excessive intake of this kind of product for a long time. Although Maruzani et al. [34] and Manaia et al. [35] suggested that a mere measurement of an antibiotic in a particular complex environment is only a component of the risk assessment, and does not reflect bioavailability, that is, the risk for transmission to humans is a function of their fitness in the environment as well as in the host, rather than only antibiotic concentrations, our study focused on the antibiotics concentration distribution, and risk size is still worth discussing. This is especially important because the exact bioavailability to humans is still poorly understood and the risk evaluation method which should be applied for considering this kind of bioavailability is also unknown, and it may be too late to act if we wait until we have concrete risk values. In a word, in this study, the RQ values of TCs at sewage outlets in the Weihe River were at a medium level (100% OTC and 85.7% of CTC samples > 0.1), which should be noted, prevented, and controlled, and related abuse should be avoided to decrease ecological risks.

## 4. Conclusions

(1) All selected antibiotics (OTC, CTC, and MC) were detected in the Weihe River and the highest concentration of the three antibiotics was in sewage outlets. In water, the concentration ranges of OTC, CTC, and MC were 1.56–87.89 ng/L, 1.07–26.78 ng/L, and 0.28–12.35 ng/L, respectively, with the average concentrations being 16.13 ng/L, 4.96ng/L, and 1.70 ng/L, respectively. Compared with other regional waters in China, the concentration of TCs in the Weihe River was at the general level, but the concentration of the sewage outlet was relatively higher. In sediment, the concentration ranges of TCs were 6.13–45.38 ng/g, 6.17–32.29 ng/g, and 4.80–29.74 ng/g, respectively, with the average concentrations being 20.60 ng/g, 14.48 ng/g, and 12.96 ng/g, respectively. The highest concentration points of the sewage outlets were S11, S6, and S19, respectively.

(2) The total contents of three tetracyclines (OTC, CTC, and MC) in water and sediment gradually decreased from the upper reaches to lower reaches, and the average concentrations of tetracycline in the middle and upper reaches were higher than those in lower reaches, largely due to the fact that two large-scale tributaries, the Jing River and the Luo River, with remittance in the lower reaches area, exhibited an obvious dilution effect for the contaminate. On the other hand, contrary to the upper or middle reaches area, the small population in the lower reaches also obviously decreased the emitted antibiotics.

(3) The risk assessment results suggested that all the sampling sites posed no high risk. In main streams, 37.5% of water samples of OTC and only 6.25% of CTC posed a medium risk. In tributaries, 38.8% and 22.2% of samples for OTC and CTC, respectively, may create a medium risk level, whilst others may a cause low risk. In sewage outlets, all the samples of OTC and 85.7% of CTC may lead to the medium risk level. Therefore, we should pay attention to the impact of tetracyclines pollutants and take corresponding preventive measures.

## Figures and Tables

**Table 1:** Residue levels of tetracyclines in the Weihe River.

TCs	Water (ng/L)				Sediments (ng/g)		
	Min	Max	Average		Min	Max	Average
OTC	87.89	1.56	16.13	OTC	87.89	1.56	16.13
CTC	26.78	1.07	4.96	CTC	26.78	1.07	4.96
MC	12.35	0.28	1.70	MC	12.35	0.28	1.70

**Table 2:** Residual levels of tetracyclines in surface water and sediments in different regions.

Samples	OTC			CTC			Regions	References
	Min	Max	Average	Min	Max	Average		
Surface water (ng/L)	1.56	87.89	16.13	1.07	26.78	4.96	Weihe River	This study
	-	361107.4	97433.8	-	68870.2	13640.9	Wangyang River	Jiang et al. [6]
	nd	72.8	44.2	nd	142.5	69.7	Tai Lake	Xu et al. [7]
	nd	8.9	-	nd	8.4		Boyang Lake	Ding et al. [14]
	nd	219.8	78.3	nd	46.7	3.6	Huangpu River	Chen et al. [19]
	22.32	49.55	31.67	nd	nd	nd	Xiang River	Fan et al. [20]
	nd	16.9	-	-	-	-	Jiulongjiang Estuary	Sun et al. [21]
	nd	13.6	2.76				Bohai Gulf	Niu et al. [22]
	5.13	22.5	11.16	nd	nd	nd	Changjiang Estuary	Yan et al. [23]
	4.60	67.56	15.79	-	-	-	Intertidal zone of Yellow River Delta	Zhao et al. [24]
Sediments (ng/g)	6.13	45.38	20.60	6.17	32.29	14.48	Weihe River	This study
	-	162673	3614.8	-	698.3	94.0	Wangyang River	Jiang et al. [6]
	nd	196.7	52.8	nd	48.5	19.0	Tai Lake	Xu et al. [7]
	0.6	18.6	6.9	nd	6.3	2.4	Huangpu River	Chen et al. [19]
	14.92	68.94	26.04	-	-	-	Bohai Gulf	Niu et al. [22]
	0.552	13.9	4.02	0.118	12	1.3	Changjiang Estuary	Niu et al. [23]
	nd	2.68	0.64				Yellow River	Zhou et al. [26]
	nd	42.2	6.69	nd	10.9	1.65	Hai River	[26]
	nd	76.6	10.87	nd	32.5	2.34	Liao River	[26]

nd refers to not detected.

**Table 3:** Toxicity data of antibiotics corresponding to sensitive species.

Antibiotics	Sensitive Species	Toxicity Type	Assessment Factor	EC_50_ (mg/L)	PNEC (ng/L)	References
OTC	*Pseudokirchneriella subcapitata*	Acute	1000	0.11	110	[36]
CTC	*M. aeruginosa*	Acute	1000	0.05	50	[6]

## Supplementary Materials

The following are available online at http://www.mdpi.com/1660-4601/15/9/1803/s1: Table S1: Sampling information; Table S2: Correlation coefficients (r^2^), recoveries (%), method detection limits (MDLs) (S/N= 3), and relative standard deviation (RSD) for three kinds of antibiotics in water and sediments; Table S3: The detailed antibiotic concentration of every sampling site in water and sediments.
