# Supplementary material for: Distribution Characteristics and Ecological Risk Assessment of Tetracyclines Pollution in the Weihe River, China
Source: Int J Environ Res Public Health. 2018 Aug 22;15(9):1803. doi: 10.3390/ijerph15091803 (PMC6174347; doi:10.3390/ijerph15091803)
Supplement: Supplementary file 1 [file ijerph-15-01803-s001.pdf]

International Journal of
Environmental Research and Public Health

Supplementary Materials

Distribution Characteristics and Ecological Risk Assessment of Tetracyclines Pollution in the Weihe River, China

Ying Li, Jie Fang, Xiaoyu Yuan, Yangyang Chen, Hongbin Yang and Xiaohua Fei

Table S1. Sampling information.

Types	Samples	Explanation
Main stream sampling sites	S1	The west sites of Baoji
	S3	The sites after the Qingjiang River flowed into the Weihe River
	S5	The sites after the Jinling River flowed into the Weihe River
	S8	The sites after the Beiqianhe River flowed into the Weihe River
	S10	The sites after the Qingshuihe River flowed into the Weihe River
	S13	Shitouhe River
	S15	Yangling Aquatic Center
	S17	The sites before the Heihe River flowed into the Weihe River
	S20	The sites before the Laohe River flowed into the Weihe River
	S23	The sites before the Fenghe River flowed into the Weihe River
	S26	The sites before the Zaohe River flowed into the Weihe River
	S29	The sites before the Bahe River flowed into the Weihe River
	S32	The sites after theJinghe River flowed into the Weihe River
	S34	The sites before the Youhe River flowed into the Weihe River
	S37	The sites before the Luofuhe River flowed into the Weihe River
Tributaries sampling sites	S2	Qingjianghe River
	S4	Jinlinghe River
	S7	Qianhe River
	S9	Qingshuihe River
	S12	Shitouhe River
	S16	Qishuihe River
	S18	Heihe River
	S21	Laohe River
	S22	Xinhe River
	S24	Fenghe River
	S27	Zaohe River
	S30	Bahe River
	S31	Jinghe River
	S35	Youhe River
	S38	Luofuhe River
	S39	Jundu
	S40	Luohe River
	S41	Gongzhuang
Sewage outlets	S6	The outlets of Baoji Wastewater Treatment Plant
	S11	Dazhangsi
	S19	The outlets of Xingping Wastewater Treatment Plant
	S25	The outlets of Xianyang Iron Bridge
	S28	The ditch of Caoyun
	S33	The outlets located at Yuchuan River
	S36	The outlets of Weinan Wastewater Treatment Plant

Table S2. Correlation coefficients (r^2), recoveries (%), method detection limits (MDLs) (S/N= 3) and relative standard deviation (RSD) for three kinds of antibiotics in water and sediments.

Analytes	r^2 of standard curve line	Recoveries (%)		MDLs (ng/L)	RSD (%)	
		River water	Sediment		River water	Sediment
OTC	0.9976	95~ 113	81~ 92	0.011	16.4	23.5
CTC	0.9987	86~ 102	76~ 89	0.012	22.8	27.5
MC	0.9988	79~ 95	62~ 80	0.028	15.0	20.0

Table sS3. The detailed antibiotic concentration of every sampling site in water and sediments.

Types	Samples	OTC		CTC		MC	
		River water (ng/L)	Sediment (ng/g)	River water (ng/L)	Sediment (ng/g)	River water (ng/L)	Sediment (ng/g)
Main stream sampling sites	S3	8.87	18.65	2.04	6.17	2.75	11.21
	S5	8.67	13.04	2.90	6.75	2.27	8.92
	S8	16.98	14.41	4.07	15.56	4.23	9.61
	S10	6.14	19.90	2.96	25.54	1.10	12.35
	S13	10.22	13.87	2.23	32.29	1.99	23.79
	S15	1.56	12.19	1.07	9.69	0.29	13.96
	S17	11.40	15.78	2.84	6.46	0.73	9.15
	S20	11.03		1.39		3.54	
	S23	13.17	15.10	2.87	12.33	1.60	17.16
	S26	14.53	21.62	3.75	13.21	0.93	29.74
	S29	17.04	28.38	5.40	17.32	0.67	14.87
	S32	9.70	19.56	3.11	12.92	0.56	23.57
	S34	8.47	31.52	3.76	7.64	2.51	7.52
	S37	7.83	20.43	2.54	9.13	0.28	12.97
Tributaries sampling sites	S2	7.01		1.71		0.94	
	S4	9.80	11.32	2.92	7.93	2.99	4.80
	S7	15.13	11.32	2.50	9.98	0.49	12.13
	S9	7.16	33.28	1.78	23.48	1.43	15.76
	S12	12.94	15.96	4.75	11.45	1.91	11.44
	S16	16.02	12.35	3.81	11.74	5.11	7.09
	S18	17.87	18.43	3.12	9.50	0.48	8.96
	S21	6.59	11.67	2.82	13.21	0.33	10.98
	S22	16.44	13.72	4.85	9.10	0.80	6.63
	S24	9.46	17.50	6.60	22.31	0.40	8.24
	S27	24.68	26.76	4.58	17.03	0.71	13.96
	S30	14.47	41.17	6.29	11.16	2.94	13.73
	S31	9.73	40.83	2.73	29.93	0.54	29.29
	S35	11.03	6.13	3.88	10.73	1.11	5.56
	S38	8.41	6.70	4.03	13.72	0.63	13.00

	S39	8.66	25.63	3.70	16.51	0.60	15.14
	S40	6.49	20.44	3.45	11.43	0.38	11.40
	S41	7.15	28.47	2.32	16.63	0.40	9.93
Sewage outlets	S6	27.71	45.38	10.83	17.84	4.82	15.53
	S11	69.86	32.68	26.78	29.60	12.35	14.07
	S19	47.83	30.84	19.12	15.28	1.06	13.50
	S25	87.89		13.94		1.29	
	S28	14.23		4.07		0.41	
	S33	21.87		10.55		0.26	
	S36	22.39		4.83		0.71	

© 2018 by the authors. Submitted for possible open access publication under the terms and conditions of the Creative Commons Attribution (CC BY) license (http://creativecommons.org/licenses/by/4.0/).
